# Parent–offspring conflict and its outcome under uni-and biparental care

**DOI:** 10.1038/s41598-022-05877-6

**Published:** 2022-02-07

**Authors:** Jacqueline Sahm, Madlen A. Prang, Sandra Steiger

**Affiliations:** Department of Evolutionary Animal Ecology, University of Bayreuth, Universitätsstraße 30, 95447 Bayreuth, Germany

**Keywords:** Behavioural ecology, Evolution

## Abstract

Conflicts over parental investment are predicted to be common among family members, especially between parents and their offspring. Parent–offspring conflict has been studied in many brood-caring organisms, but whether its outcome is closer to the parental or offspring optimum is usually unknown, as is whether the presence of a second parent, a caring male partner, can affect the outcome. Here, we manipulated the initial brood size of single and paired female burying beetles to examine how many offspring are necessary to maintain parental care in the current brood. We found that mothers continued to invest in small broods even if their reproductive output would have been higher if they had discontinued their care and produced a second brood instead. Consequently, our data suggests that the offspring have the upper hand in the conflict. However, our results further show that paired females laid a second egg clutch more often and produced more offspring than single females, suggesting that the presence of a male partner shifts the conflict outcome towards the parental optimum. This latter result not only is a novel aspect of parent–offspring theory, but also represents an additional factor that might explain the evolution of biparental care.

## Introduction

Family life involves cooperation and conflicts between family members. One central conflict is the dispute between parents and offspring over parental investment^1–6^. This potential disparity in the optimum level of parental investment arises because of asymmetries in relatedness, i.e., an offspring is more related to itself than to any of its current or potential future siblings, whereas parents are equally related to all their offspring. Consequently, each offspring is selected to demand more investment than parents should provide. Two different sorts of parent–offspring conflicts are known from theory, namely the intra-brood conflict, in which parents and offspring battle over resource allocation among members of the current brood, and the inter-brood conflict, in which disagreement arises concerning resource division between current and future offspring, since investment in current offspring should reduce the amount of resources available for future offspring^1,4,7^. However, a central question is still in whose favor the conflict is resolved.

Although there are few studies that have found evidence for a parent–offspring conflict to occur in nature^8–11^, whether the outcome of the conflict is closer to the offspring’s or the parents’ optimum is usually unknown^12^. The reason for this lack of information is that the determination of the investment optima for parent and offspring is a difficult task. In some cases, parents might obviously have the upper hand. For example, offspring are not able to influence the amount of nutrition that is provided into their eggs^9^. However, in cases in which parents and offspring interact after birth, offspring might be capable of manipulating parental physiology in such a way that care is prolonged or increased in the current brood at the expense of future reproduction^13,14^. For example, in a variety of mammals, the continued suckling of young causes a temporary infertility in mothers^15–17^, and in honey bees, larval begging pheromones have not only been shown to positively affect food provisioning, but also to inhibit egg development in nursing workers^18,19^. Although these examples illustrate that offspring might have the potential to affect the trade-off between investment in current offspring and the parent’s expectation of future offspring, it is currently unclear whether they are indeed able to bias the conflict outcome toward their own optimum. A manipulation of parental investment might be achieved by using exaggerated begging signals^1^ or, as suggested by Mas and Kölliker^13^, by solicitation pheromones with a priming effect on maternal physiology.

Surprisingly, at present, we also lack data about whether the outcome of the battle is different under uniparental versus biparental conditions. In nature, the most common form is female uniparental care, but also male and biparental care can occur. There are even species, in which the family composition can vary from brood to brood. Especially in systems in which care is flexible, and in which uniparental care occurs alongside biparental care, offspring might not have the ability to affect the physiology of both parents to the same degree as that of a single parent. For example, in the presence of a helping male, females might reduce their amount of contacts with offspring, resulting in less offspring control over maternal physiology and reproductive behavior.

Since we are still far away from understanding all the facets of intrafamilial conflicts and how they impact the evolution of family life, we studied parent–offspring conflict by using the burying beetle *Nicrophorus vespilloides* as a model organism. Specifically, by analyzing more than 500 families, we tested (1) whether an interbrood conflict exists, (2) whether the outcome of the interbrood conflict is closer to the parents´ or the offspring optimum and (3) how the outcome is affected by the presence of a male partner. *N. vespilloides* is a particularly valuable study system to address these questions because, first of all, an earlier preliminary study has provided some indications of an interbrood conflict^20^, and secondly, both biparental care and female uniparental care occur in natural populations of this species^21,22^.

Burying beetles reproduce on small vertebrate cadavers that serve as a food source for their offspring^23–26^. Upon finding a carcass, parents bury it within the soil, thereby removing fur or feathers, rolling it into a ball-shape, and treating it with antimicrobial secretions to manipulate the microbiome and to reduce decay^27–33^. The parents then cut a feeding cavity into the carcass in which the developing larvae are not only fed by the parents, but can also self-feed from the resource^24–26,34–36^. Larvae are known to beg for food by raising their head toward the parent while waving their legs^36,37^. Both parents respond to begging, but females engage in a higher rate of food provisioning than males^21,38,39^. Since a vertebrate cadaver is a rare but very valuable and highly contested resource, burying beetle parents are expected to make careful decisions about brood sizes to optimize the exploitation of the resource. Due to, for example, egg predation or hatching failure, the initial brood size on a carcass can be small. In such a situation, females have been observed to resume egg laying and produce a second clutch on the same cadaver^40^. However, although investing in a second clutch might enhance the overall reproductive output of the parents, a preliminary study^19^ suggests that this has negative effects on the offspring of the first brood, because mothers that resume egg laying have been observed to close the feeding cavity^24^. This helps to preserve the carcass and slows down the deterioration of resource quality^35^, but also implies that they discontinue to feed their current offspring. Eggert and Müller^24^ reported in their review that closing the feeding cavity sometimes even results in the death of the larvae, presumably because they suffocate. Consequently, there might be a parent–offspring conflict over the production of a second clutch and offspring might have evolved mechanisms to inhibit maternal egg production and to promote parental care. In fact, since offspring on a specific carcass are often of mixed paternity the genetic asymmetry within families is stronger leading to higher levels of conflict than in full-sib families^22,41^. However, whether a conflict really exists, and which party wins the dispute under uni- and biparental conditions is currently unclear.

To address these gaps in our understanding of parent–offspring conflict, we conducted two experiments that were specifically designed to test the following predictions. We first of all predicted that females would be able to produce a second clutch on the same cadaver and that the probability to lay eggs would decrease with initial brood size and increase with carcass size. We furthermore predicted that there would be a parent–offspring conflict over the production of a second clutch. We expected that females that were confronted with a small brood should benefit from producing a second clutch, but offspring should suffer from such a response, as females then close the feeding cavity and discontinue to care. Our experimental design also allowed us to evaluate whether the outcome of the conflict was closer to the parents’ or the offspring optimum and whether the outcome differed between uni- and biparental families. Based on the hypothesis of Mas and Kölliker^13^, we predicted that larvae would be able to influence maternal reproductive physiology and behavior and bias the outcome towards their own interest. However, we also predicted that in the presence of a caring male partner, the outcome would shift in the direction of the parental optimum. Females have been shown to decrease their provisioning rate when caring with a male^38^. Because of the reduced mother–offspring interactions in biparental families, offspring might not have the power to affect their mother’s reproductive physiology in such a way as in uniparental families.

## Material and methods

### Origin and husbandry of the beetles

We used virgin beetles from an outbred laboratory population kept at the University of Bayreuth. The beetles were the 3rd-5th generation of beetles descending from wild-caught beetles collected in a forest in Bayreuth, Germany, during the summer of 2018. Prior to the experiment, the beetles were kept in small plastic boxes (10 × 10 × 6 cm) filled with moist peat. The beetles were maintained in a climate chamber at 20 °C under a dark: light cycle of 16:8 h and were fed with sliced mealworms twice a week. At the start of the experiment, beetles had either an age of 20 (N = 288) or 30 days (N = 288).

### General experimental designs and procedures

To test our predictions in the context of parent–offspring conflict we performed two experiments. Both experiments were conducted in climate chambers at 20 °C. In the first experiment, we used a 6 × 3 × 2 factorial design in which we manipulated the initial number of larvae provided (0, 1, 2, 3, 5, or 10 larvae), the carcass size (mice carcasses of approx. 5 g, 10 g, and 20 g), and the absence or presence of a male partner (i.e., single, and paired females). The treatment, in which parents were initially not provided with any larvae served as a control group. In this group, no parent–offspring conflict could occur and consequently, larvae could not influence a female’s decision to lay a second clutch. For each treatment group, we set up 16 replicates, resulting in an overall sample size of N = 576. Our final sample size was reduced to N = 544, because of the failure of 32 beetles to lay a first egg clutch.

We set up each pair by placing an unrelated virgin male and female in a plastic box quarter-filled with moist peat (10 × 10 × 6 cm). Pairs stayed together for 72 h to allow repeated mating in order to ensure a sufficient supply of sperm for the fertilization of eggs. In the case of single female trials, we removed the males after the mating period. To test paired females, males were kept with their partner during the entire treatment. After the initial mating period, single and paired females received access to a freshly thawed mouse carcass to initiate breeding. Therefore, a larger amount of moist peat was added to the boxes. Forty-eight hours later, the carcass and beetles were placed into a new, similarly sized plastic box filled with moist peat. This procedure ensured that the eggs were separated from the parents, and that the larvae hatched in isolation. The old container was checked for newly hatched larvae every 4 h day and night for overall 48 h. Hatched larvae were pooled together in a Petri dish on a wet filter paper. Larvae were then randomly assigned to the different treatment groups. As parents kill any larvae that arrive on the carcass before their own larvae are expected to hatch^42^, we only provided parents with a brood once their larvae had hatched. In this species, parents do not distinguish between unrelated foster larvae and their own offspring^42^ making it possible to provide parents with larvae of mixed parentage (see e.g.^43–45^).

After the parents had received a brood of a certain size, we checked the broods every 6 h for 72 h, and then we increased the time interval to 8 h for another 72 h. After 6 days, we checked the boxes every 12 h until larvae dispersed from the carrion resource. During the observation periods, we noted whether females had laid a second clutch or not, whether the cavity was open or closed (i.e., whether the females had discontinued to care for broods or not), and whether larvae within a closed feeding cavity were alive or not. Furthermore, as soon as the larvae dispersed for pupation, we recorded the brood size. After dispersal, we transferred the larvae to a new plastic container filled with moist peat for pupation. After eclosion, the number of emerging adults was determined for each brood.

In the second experiment, we provided single females with a freshly thawed mouse carcass of a standardized size (7.5–12.5 g) and small initial broods of varying sizes (0, 1, 2, or 3 larvae). The experimental procedure was similar as described above. However, in our second experiment, we not only counted the number of dispersing larvae, but also determined their mass. We also weighed the females before and after the breeding event to test whether laying a second clutch had a negative effect on the females’ weight change during breeding and therefore body condition. In total, we tested 15 single females per treatment (N = 60). Due to the failure of three beetles to a lay a first egg clutch, the final sample size was reduced to N = 57.

### Statistical analyses

All data were analyzed and plotted using R version 3.5.1. In a first step, we analyzed which factors influenced a female’s decision to lay a second clutch. Therefore, we used the data of our first experiment and conducted a generalized linear model (GLM) fitted with a binomial error structure. As predictors, we included initial brood size (as continuous variable), carcass size, and the presence or absence of a male partner. Since the full-factorial design showed no significant effect of all possible interaction terms between the three factors, we excluded the interaction terms from our analysis.

In a second step, we determined whether there is a parent–offspring conflict over the production of a second clutch, we evaluated the fitness of parents and offspring when females produced a second clutch or not. In our first experiment, we determined whether and how often parents closed the feeding cavity when producing a second clutch and whether the fitness of the current offspring was affected, i.e., whether they died within the closed feeding cavity. To evaluate the fitness of parents, we calculated Wilcoxon rank-sum tests to compare the overall number of dispersing larvae and the number of emerging adults produced by females that laid a second clutch and those that continued to care for their current brood. Using the data from our second experiment, we further investigated the average weight of a larva at dispersal as a fitness parameter for offspring and the and carcass use efficiency as a maternal fitness parameter. Carcass use efficiency, which is calculated by dividing the brood mass at dispersal by the initial carcass mass, indicates how much carrion biomass a female is able to convert into offspring biomass^46,47^. We used Welch’s t-tests (because of unequal variances) to compare the mean larval weight and the carcass use efficiency of females that laid a second clutch and those that did not. Furthermore, as an additional fitness indicator for females, we evaluated their body weight change during breeding. To this end we used a by a two-sample t-test (because of equal variances) in order to compare the weight change of females that laid a second clutch with those that did not.

In a third step, we evaluated whether the outcome of parent–offspring conflict was closer to the parents’ or offspring optimum. Therefore, we used the data from our first experiment and determined the smallest initial brood size that was necessary to prevent more than 50% of the females from laying a second clutch and that triggered them to continue to invest in the current brood. We refer to this brood size as the ‘larval tipping point’. Using a Wilcoxon rank-sum tests, we then compared the number of larvae raised at the tipping point with the number of offspring that parents from the control group (treatment in which initial brood size was zero) were able to produce. If the number of larvae that was able to suppress egg laying was significantly smaller than the number of larvae that females of the control group were able to raise, this would indicate that the parent–offspring conflict was resolved closer to the offspring than the parents’ optimum. The rationale behind this argument is that the mothers’ fitness would have been greater, if they had laid a second clutch instead of continuing to invest into the current brood. In the control, the mothers’ decision to lay a second clutch was not influenced by the presence of larvae. We ran the tests separately for single and paired females to evaluate whether the outcome of parent–offspring conflict differed under uni- and biparental conditions. Furthermore, we calculated a binomial GLM to test for an effect of a male partner on the care strategy of females (i.e., whether they closed the feeding cavity or not). Finally, we calculated a quasi-Poisson GLM (to correct for overdispersion) in order to investigate the effects of male presence, initial brood size, and carcass size on the number of dispersed larvae.

## Results

### Do females lay a second egg clutch, and which factors influence its production?

**Figure 1:**
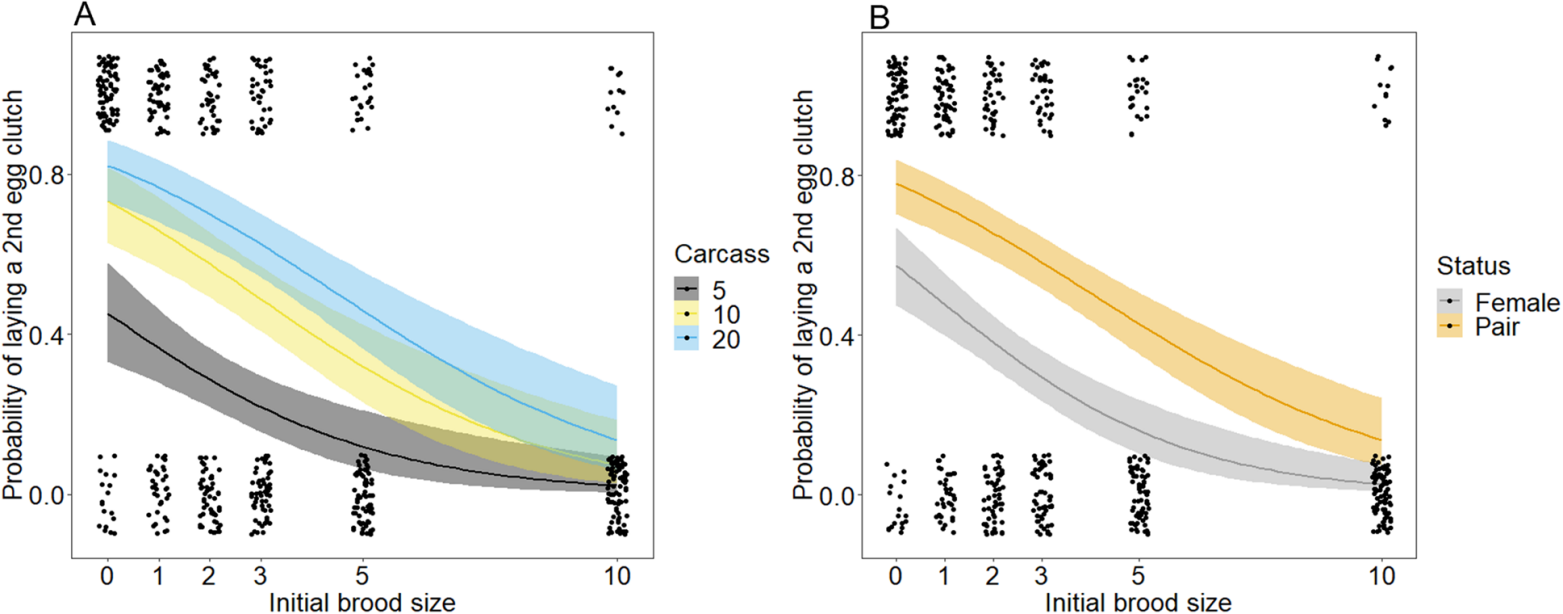
Relationship between the probability of *N. vespilloides* females to lay a second egg clutch and initial brood size (**A**) on three different carcass sizes and (**B**) when breeding with a male partner or alone. The dots represent the original data, the lines represent the calculated regression lines and their respective 95% CI.

Females responded to our brood size manipulation by laying a second egg clutch in 236 of 544 trials. As predicted, the egg-laying strategy was affected by the initial brood size and by the carcass size: the beetles’ probability of laying eggs decreased with an increasing initial brood size and a decreasing carcass size (Table 1; Fig. 1A). The presence of a male partner also affected the egg-laying strategy of *N. vespilloides*. Single females showed a lower probability for laying a second egg clutch than females caring with a male partner (Table 1; Fig. 1B).

**Table 1:** Summary of models for the effects of initial brood size, carcass size, and the male partner on the probability of producing a second clutch and the number of dispersing larvae.

Predictors	Egg-laying probability			Number of dispersing larvae		
	F	df	p	F	df	p
Initial brood size	98.33	1	**< 0.001**	0.01	1	0.92
Carcass size	25.34	2	**< 0.001**	17.39	2	**< 0.001**
Male partner	34.96	1	**< 0.001**	5.22	1	**0.02**

Significant values are in bold.

### Is there a parent–offspring conflict over the production of a second clutch?

**Figure 2:**
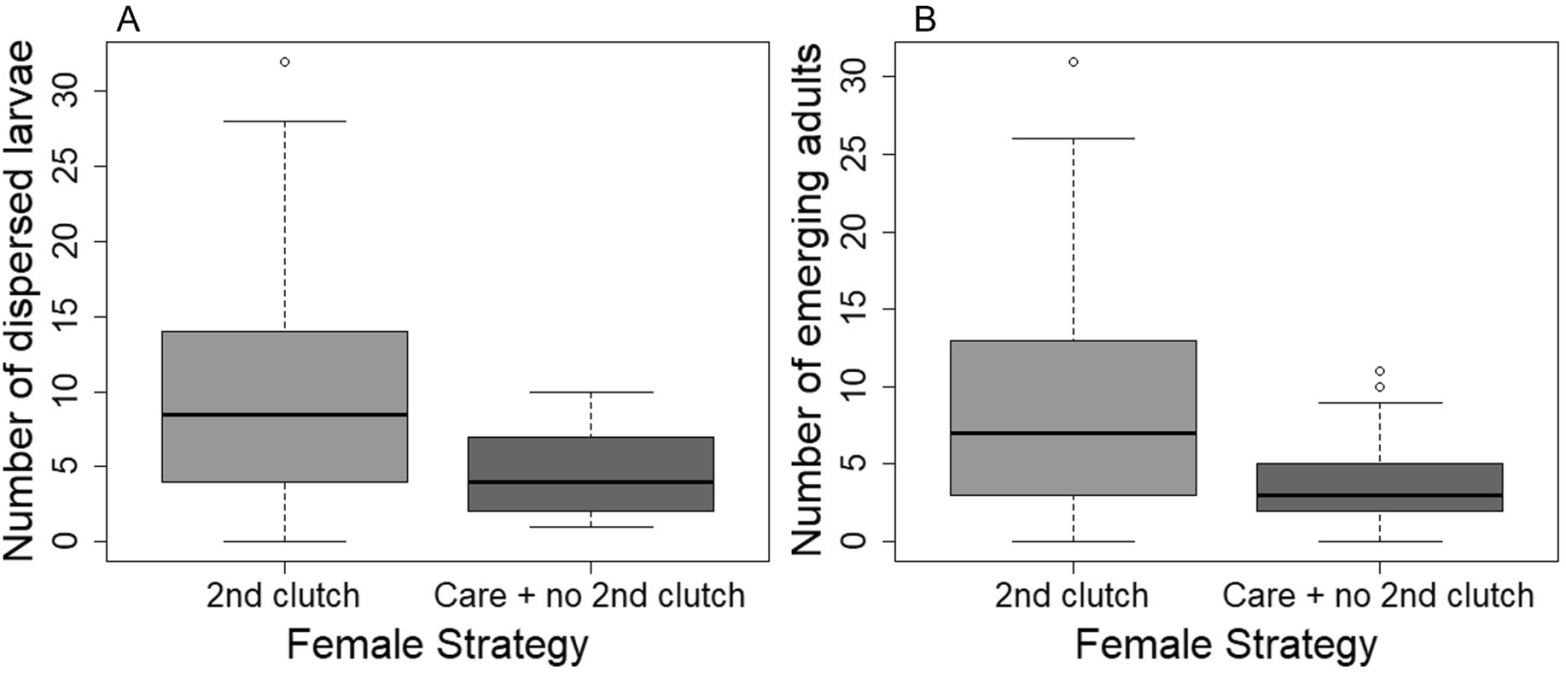
(**A**) Number of dispersed larvae and (**B**) emerging adults of those females that did not produce a second clutch but continued to care for the initial brood and those females that produced a second clutch.

**Figure 3:**
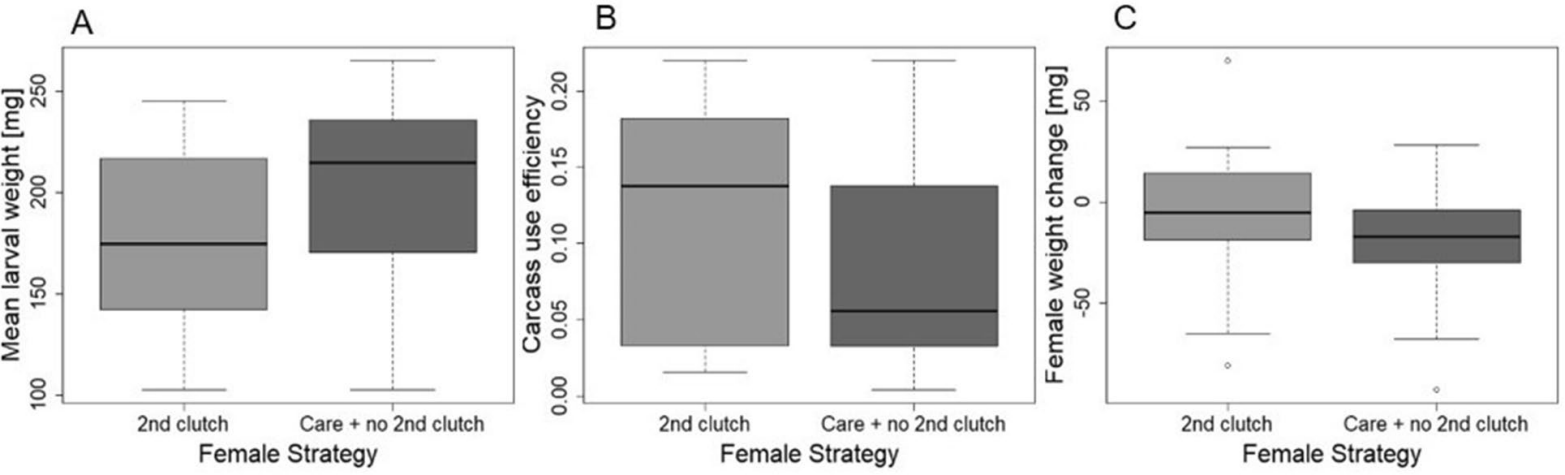
(**A**) Mean larval weight, (**B**) carcass use efficiency (total brood mass/carcass mass), and (**C**) body weight change during breeding of those females that did not produce a second clutch but continued to care for the initial brood and those females that produced a second clutch.

As we predicted, when females responded to small broods by laying a second clutch, they frequently closed the feeding cavity (N = 145). In 37 cases, the entire brood even died. However, whereas the production of a second clutch had a negative impact on offspring fitness, it was beneficial to females, as they were able to produce an overall higher number of dispersing larvae (Wilcoxon rank-sum test; W = 14,781, p < 0.001; Fig. 2A) and a higher number of offspring surviving to adulthood (Wilcoxon rank-sum test; W = 15,096, p < 0.001; Fig. 2B) than mothers that did not lay a second clutch but that continued to care for the current offspring. The finding of antagonistic fitness effects between parent and offspring was further substantiated by our second experiment, which considered not only the number of dispersing larvae, but also their mass. Larvae of mothers that did not lay a second clutch but went on to care for their initial brood were characterized by a higher mean mass than the offspring of mothers that produced a second clutch (Welch-two-sample t-test; t = 2.19, df = 46.46, p-value = 0.03; Fig. 3A). However, mothers that laid a second clutch showed a higher carcass use efficiency than mothers that did not resume egg laying (Welch-two-sample t-test, t = 2.07, df = 44.39, p = 0.04; Fig. 3B). Our second experiment also showed that the production of a second egg clutch did not negatively affect the body weight of the females, as we could not find a difference in the overall weight change during the reproductive event between females that laid a second clutch and those that did not (two-sample t-test, t_50_ = − 1.37, p = 0.17; Fig. 3C).

When looking at the different female ‘strategies’ in more detail, we found that, in the majority of cases (405 out of 544; Supporting information Fig. S1), active care for the initial brood and egg production were mutually exclusive events: Females either started to produce a second clutch and closed the feeding cavity of the carcass, or they accepted the initial brood without laying a second clutch. Surprisingly, we found that 91 females laid a second clutch but did not close the cavity (Supporting information Fig. S1), suggesting that females were able to produce further eggs, while caring for the current brood. However, when we analyzed the total number of dispersed larvae, we found that females that laid eggs but did not close the cavity raised a lower number of offspring than females that closed the cavity and laid a second clutch (Wilcoxon rank sum test, W = 7235, p < 0.001; Supporting information Fig. S2A). The same was true for the number of surviving offspring to adulthood (Wilcoxon rank sum test, W = 7366, p < 0.001; Supporting information Fig. S2B).

### Is the conflict outcome closer to the parents’ or the offspring optimum and does it differ between uni- and biparental families?

**Figure 4:**
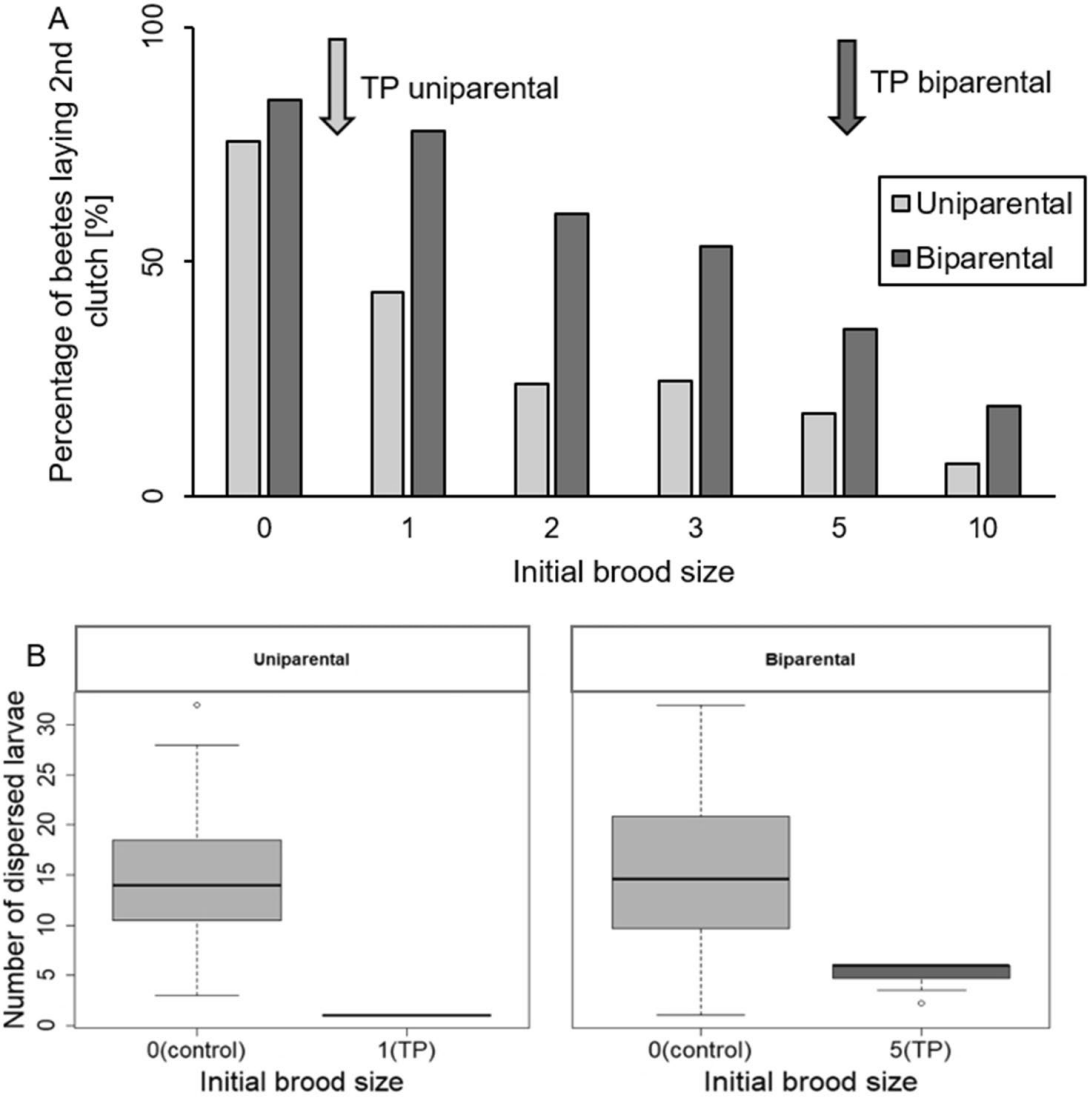
(**A**) Bar plot showing the tipping points of females in uniparental (gray) and biparental condition (dark grey). Arrows in respective colors mark the tipping points (= TPs), i.e., the initial brood size at which less than 50% of females resumed egg laying. Note that at both tipping points, the proportion of females that did not produce a second clutch was significantly smaller than in the respective control groups (i.e. the treatment in which brood size was zero and females did not receive any larvae; uniparental females: *Chi*^2^ = 9.70, p = 0.002, biparental females: *Chi*^2^ = 22.41, p < 0.001). (**B**) Boxplots showing number of dispersed larvae of females of the control groups and females that did not produce a second clutch at the respective tipping points.

**Figure 5:**
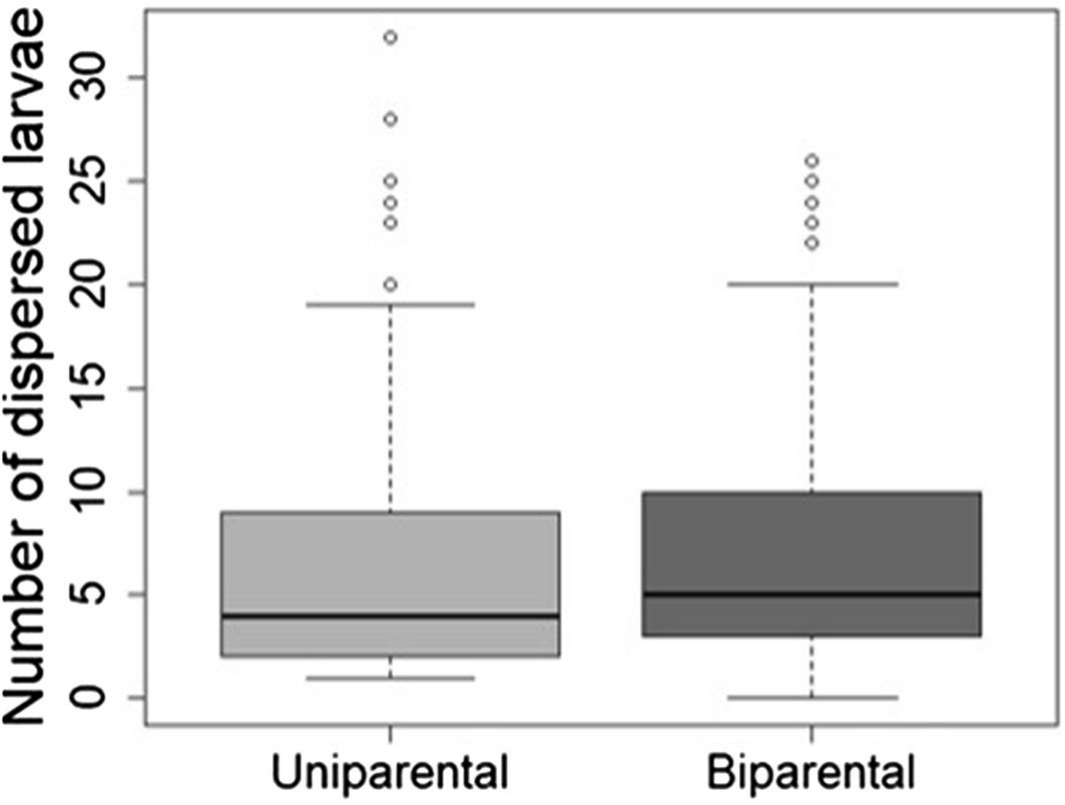
Boxplots showing the number of dispersed larvae produced by females under uni- and biparental conditions.

To examine in which direction the parent–offspring conflict is solved in uni- and biparental families, we first determined the ‘larval tipping point’, from which onward, the majority of females decided to invest in the current brood instead of producing further offspring. We found a tipping point of one larva for single caring females and five larvae for biparental females (Fig. 4A). Hence, a larger number of larvae were necessary to suppress egg laying under bi- than uniparental condition. In fact, not only did biparental females show a higher probability of laying a second clutch (Table 1, Fig. 1B) and of closing the cavity (GLM, ${\mathrm{Chi}}_{\mathrm{1,453}}^{2}$ = 18.66, p < 0.001), but these decisions also led to a larger number of dispersing larvae than seen in single females (Table 1, Fig. 5). However, under both family social conditions parents that did not produce a second clutch at their respective tipping point raised less larvae than parents of the control group (i.e., parents that were initially not provided with any larvae; uniparental: Wilcoxon rank sum test, W = 513, p < 0.0001; biparental: Wilcoxon rank sum test, W = 823.5, p < 0.0001; Fig. 4B). Hence, these results suggest that the conflict over the production of a second clutch is resolved closer to the offspring optimum.

## Discussion

Parent–offspring conflict is thought to be a key element of family life. However, empirical evidence is scarce, and it is usually unknown, which party wins the dispute^12^. Here, we found evidence for a parent–offspring conflict over resource allocation between current and future offspring. When confronted with a small brood, females frequently responded by laying a second clutch. However, the investment in a second brood caused a simultaneous increase in parental fitness and a decrease in offspring fitness: Females that stopped investing in their first brood and resumed egg laying had a higher fitness, but the current offspring suffered from discontinued feeding. This suggests a disagreement between parents and offspring over the production of a second brood. Our study furthermore suggests that the outcome of the conflict is closer to the offspring optimum under uniparental care but is shifted in the direction of the parental optimum in the presence of a male partner. Hence, the offspring might be capable of influencing the maternal investment decision, but this capability might be negatively affected by the presence of a second parent.

Vertebrate carrion is a valuable nutrient-rich resource, but corpses suitable for the reproduction of *Nicrophorus* are thought to be rare and difficult to monopolize and hence should be converted efficiently into offspring biomass^24^. When the mortality of eggs or larvae is high, such that fewer offspring are present than the carrion can support, female burying beetles have been observed to produce a second clutch^40,48,49^. We could confirm these previous findings, since nearly half of the female *N. vespilloides* tested responded to our resource and brood size manipulation by laying a second egg clutch. As predicted, we found that the probability of resuming egg laying increased with resource size and decreased with initial brood size. This suggests that females can assess brood and cadaver size and enhance the usage of the valuable resource by producing additional eggs. Indeed, our results revealed that females that responded to a low offspring-carrion ratio by resuming egg laying produced a higher number of dispersing offspring and converted more carrion food into offspring biomass than those that did not.

Optimal clutch size decisions have been studied intensively over the last few decades^50–54^. Many insects oviposit on food patches that represent finite resources and, similar to burying beetles, have been shown to have the ability to adjust the number of eggs laid according to the size and quality of the patch^55–57^. However, when elaborate parental care is involved, the optimal oviposition strategy can differ for parents and offspring, leading to a parent–offspring conflict^11,58,59^. Our results indeed highlight that the production of a second clutch enhanced maternal fitness while simultaneously reducing the fitness of the current offspring. Females that effectively resumed egg laying closed the feeding cavity in which the larvae reside and in several of these cases broods died. Eggert and Müller^24^ suggested that the larvae suffocate in such a situation, their death being an accidental consequence of the parents’ attempt to maintain resource quality for future offspring (see also^35^). Although the initial offspring suffered in our study, mothers produced more dispersing larvae and used the carcass more efficiently compared to mothers who left the feeding cavity open and continued to care for the first brood. Conversely, mothers that invested only in their first brood showed a lower fitness, but their offspring profited from prolonged care, since our second experiment revealed that those larvae had a higher average mass at dispersal. Consequently, we have found evidence for an inter-brood conflict over parental investment. Although the occurrence of an inter-brood conflict has been predicted by theory, it has seldom been shown in natural systems due to the experimental difficulties of demonstrating divergent fitness optima for parents and offspring. A study of Kölliker et al.^11^ also found evidence for an inter-brood conflict by examining the trade-off between offspring fitness and a parent's ability to produce a second clutch in earwigs *Forficula auricularia*. Interestingly, in contrast to our study, they found a conflict at the egg stage but not after the nymphs have hatched. Hence, our study contributes to our understanding of family living by empirically showing that parent–offspring conflict over the production of a second brood can also occur after the birth of offspring.

Our fitness analysis indicates that the outcome of the parent–offspring conflict is closer to the offspring optimum, since fewer larvae were necessary to inhibit oviposition than the carcasses would potentially support. In fact, in uniparental families one larva was already enough to suppress egg laying in more than 50% of the females and in biparental families five were required. In both cases, parents raised less larvae than parents from the control group, in which the first brood was removed and the majority of females resumed egg laying. Consequently, females would have benefited from producing a second clutch instead of continuing to care for small broods. The result is especially surprising as parents are thought to be more powerful than offspring and should be able physically to dominate their young^3,60^. But why do parents provide more care than they should? One possible explanation is that the investment optimum of females is closer to the offspring’s interest than our fitness analysis suggests. When confronted with a small brood, females can optimize the number of larvae raised and hence the amount of carrion they convert into offspring by laying a second clutch. However, females might use a small brood as an indicator for a harsh environment (e.g., high risk of egg predation), low body condition or lack of sperm and hence, do not anticipate a larger second brood. Furthermore, our analysis only considers reproduction on the current carcass, and not future breeding success on new carcasses. Preserving the carcass for a second brood, which involves combatting microbial competitors and pathogens, might result in high energetic or physiological costs leading to a reduced future reproductive value and a lower lifetime reproductive success. Although this explanation seems plausible, some factors speak against it. First, we found no differences in body mass changes between females that produced a second clutch and those that had not, indicating equal energetic costs of both strategies. Secondly, females can produce up to five replacement clutches, if the first clutches are removed^40^, which also argues against high costs.

An alternative explanation for our results might be that manipulative offspring signals are involved. Parent–offspring conflict is thought to drive the evolution of begging signals. Those signals might honestly reflect the need of the offspring, but also might have the potential of manipulating parents into investing more than they should^3,13,61–65^. Although such manipulative agents have been considered to be evolutionary unstable (see also^66–68^ for a debate about queen pheromones either acting as honest signals or manipulative chemicals), theoretical considerations suggest that offspring might indeed produce manipulative signals. Mas and Kölliker^13^, for example, have proposed a hypothetical mechanism by which offspring-derived primer pheromones influence the hormone system of mothers’ thereby suppressing egg production and maintaining parental care in the current brood. Although a tactile begging signal exists in burying beetles^36,69,70^, it is currently unknown whether offspring also produce a chemical signal that influences maternal physiology and behavior. We know, however, from a previous study that larvae are able to affect maternal hormone titer and reproductive behavior^48^. Furthermore, burying beetle adults are known to produce a range of chemical signals that serve to coordinate mating and breeding, and hence, larvae probably also communicate chemically^45,48,71–75^. In agreement with our hypothesis, recent experiments indicate that mothers also respond to cues other than tactile begging behavior to adjust their provisioning rate^76,77^. Like us, the authors proposed that chemical cues play an important role in mediating parent–offspring interactions in burying beetles (see also^78^). We advocate that future studies investigate the existence and the effect of potential begging pheromones in parent–offspring associations in burying beetles.

Models of conflict resolution either predict that investment levels lie somewhere between parent and offspring optima or close to the parents’ optimum^12^, which appears to contradict our findings. However, there are also observations of other family systems, in which offspring seem sometimes to have the upper hand in the battle. For example, there is evidence that during mammalian pregnancy, embryos are capable of influencing maternal blood sugar levels and therefore maternal investment^79^. As a consequence, some mothers suffer from diabetes, but the offspring benefit from gaining more weight. In future work, it will be important to evaluate the outcome of parent–offspring conflict in a range of different species to obtain a more profound picture on how and in which direction the conflict is resolved.

A further key finding of our study is that the outcome of the conflict is shifted slightly towards the parental optimum in the presence of a male partner: more larvae were necessary to suppress the production of a second clutch in biparental than uniparental females. Biparental females were more likely to resume egg laying and, in consequence, produced a larger number of dispersing larvae than uniparental ones. Since males contribute to offspring feeding, mother–offspring interactions are slightly reduced in the presence of males^38^, and hence, offspring might have less opportunity to affect maternal reproductive physiology and behavior than in the absence of males. Interestingly, *N. vespilloides* offspring have been shown to preferentially beg towards females^80^. In view of our findings, such a discrimination between female and male parents should be highly adaptive, since only the interaction with mothers can affect maternal reproductive physiology and ensure that the females do not stop caring and resume egg laying.

Another possibility is that the presence of a male partner triggers a divergence of the maternal interest from the offspring interest rather than causing a shift of the conflict’s outcome towards parents. The lack of hatchlings or the presence of only a few of them arriving at the carcass might serve as a cue for unfertilized eggs and sperm depletion, and a resumption of egg production might therefore be more advantageous after additional matings and sperm transfer (i.e., in the presence of a male). In agreement with this hypothesis, Sakaluk et al.^81^ found that widowed females were less likely to produce a replacement clutch once the first clutch had been removed. It is also possible that with the male’s help in carcass maintenance, males reduce the costs of producing a second clutch for females^82^. However, this explanation only holds when prolonged carcass maintenance entails substantial costs. Fruitful directions for future studies would be to test whether sperm depletion can play a role, and to assess whether prolonged investment in carcass maintenance entails higher costs for uniparental than biparental females.

Regardless of the mechanism behind the shift in the conflict outcome, our findings emphasize that we need to consider number of parents when studying parent–offspring conflict. Furthermore, our study highlights that males benefit from a prolonged association with the family. Previous studies found that offspring thrived equally well when reared by male–female pairs or single females on same sized cadavers in the absence of competitors^83–88^. The question of why males remain with females on the carcass for extended periods remained largely unsolved (but see^89^ for possible synergistic effects of biparental care). One factor that might have promoted the evolution of extended male residency is that males benefit from carrion consumption^90^. However, our current study has revealed an additional important aspect that might explain the evolution of biparental care. In a case in which, for example, many eggs fail to hatch the presence of a male ensures the better utilization of the carcass, since paired females have a higher tendency to produce a second clutch, leading to a larger number of dispersing offspring.

To conclude, our study has found evidence for parent–offspring conflict over the amount of parental investment. Whereas parents benefit from the production of a second clutch when initially confronted with a small brood, their current offspring suffer from this decision. Furthermore, our study indicates that the conflict outcome is closer to the offspring than the parents’ optimum. Future studies will help to understand whether offspring-derived chemical signals can influence maternal physiology and behavior and target the trade-off between the care for current offspring and the production of new eggs. Finally, our study advances our understanding of parent–offspring conflict by demonstrating that the presence of a male partner can alter the conflict outcome either because investment levels shift in the direction of the parental optimum or because of an increased deviation between maternal and offspring interests. Such an effect on the conflict outcome could be revealed because biparental care is facultative in burying beetles. It would be interesting to investigate, whether parent–offspring conflict is also affected by the presence of a partner in other family systems with facultative biparental care. We also want to highlight that our results exposed substantial variation in the conflict outcome among families. As previously advocated by Kilner and Hinde^12^, future studies in the field of parent–offspring conflict not only should focus on population averages but should also acknowledge individual differences. Burying beetles represent an ideal model system for studying such individual variation.

## Supplementary Information

Supplementary Figures.

## Data Availability

Data will be submitted to DRYAD. The doi for our data is: 10.5061/dryad.63xsj3v3x.
