# Author Correction: Parent–offspring conflict and its outcome under uni-and biparental care

**DOI:** 10.1038/s41598-022-07649-8

**Published:** 2022-03-02

**Authors:** Jacqueline Sahm, Madlen A. Prang, Sandra Steiger

**Affiliations:** Department of Evolutionary Animal Ecology, University of Bayreuth, Universitätsstraße 30, 95447 Bayreuth, Germany

Correction to: *Scientific Reports* 10.1038/s41598-022-05877-6, published online 07 February 2022

In the original version of this Article, Sandra Steiger was incorrectly indicated as a corresponding author. The correct corresponding author for this Article is Jacqueline Sahm. Correspondence and request for materials should be addressed to Jacqueline.Sahm@uni-bayreuth.de.

The original Article has been corrected.

