# Supplementary material for: Parent–offspring conflict and its outcome under uni-and biparental care
Source: Sci Rep. 2022 Feb 7;12:1999. doi: 10.1038/s41598-022-05877-6 (PMC8821718; doi:10.1038/s41598-022-05877-6)
Supplement: Supplementary file 1 — Supplementary Figures. [file 41598_2022_5877_MOESM1_ESM.docx]

**Supporting Information**

**Parent-offspring conflict and its outcome under uni-and biparental care**

Jacqueline Sahm^1^, Madlen A. Prang^1^, Sandra Steiger^1*^

**Figure S1:** Proportions of different strategies used by females when confronted with small broods. Females produced a second clutch or did not and closed the cavity or did not.


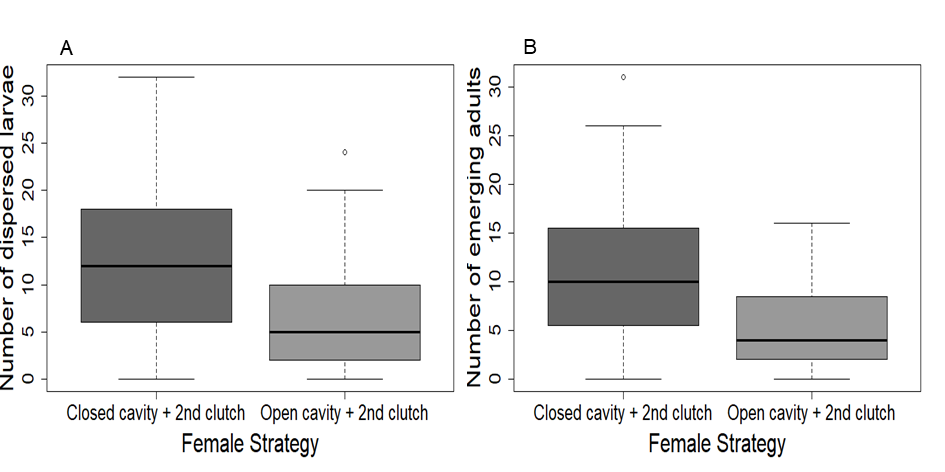


**Figure S2**: (A) Number of dispersed larvae and (B) emerging adults of females that produced a second clutch while leaving the feeding cavity open or closing the cavity.
